# Re-evaluating breast malignant pleural effusion: toward evidence-based, precision-aligned care with organoids

**DOI:** 10.3389/fbioe.2026.1750983

**Published:** 2026-05-13

**Authors:** Gavin R. Oliver, Kshama Jaiswal, W. Roy Smythe, Carlton C. Barnett

**Affiliations:** 1 Xilis, Inc., Durham, NC, United States; 2 University of Utah School of Medicine, Salt Lake City, UT, United States; 3 University of Colorado School of Medicine, Aurora, CO, United States

**Keywords:** indwelling pleural catheter, malignant pleural effusion, next-generation sequencing, organoid, pleurodesis, standard-of-care

## Abstract

Breast cancer–associated malignant pleural effusion (MPE) is a common and debilitating manifestation of advanced disease, yet current management is largely limited to indwelling pleural catheters and chemical pleurodesis and offers only transient palliation without addressing the underlying tumor biology. We propose that integrating patient-derived organoid modeling of pleural tumor cells with characterization via technologies like next-generation sequencing could shift MPE care from symptom management toward precision intervention. Organoid-based drug testing enables *ex vivo* evaluation of local therapeutic agents, including intrapleural chemotherapy, immune modulators, and bispecific antibodies, while paired genomic profiling may reveal actionable resistance pathways unique to pleural metastases. Together, these approaches could identify rational, localized combination therapies that improve local control, reduce effusion recurrence, and ultimately extend survival. By coupling functional and molecular analyses directly to the pleural compartment, we envision a translational framework that redefines breast MPE from a purely palliative condition to one amenable to mechanism-driven, patient-tailored therapy.

## 1 Introduction

Breast cancer is the most commonly diagnosed cancer in women and the most fatal. Worldwide, approximately 2.3 million women per year are diagnosed with this biologically unique and frequently aggressive malignancy and approximately 670,000 succumb annually (Filho et al., 2025). While the risk of breast cancer increases with age and the peak age of diagnosis in Western countries is 60, those affected may also be premenopausal (Winters et al., 2017) with significantly reduced quality of life (Vrancken Peeters et al., 2025). Despite advances in treatment, metastatic disease is generally considered incurable (Cohn et al., 2025).

Malignant pleural effusion (MPE) is the accumulation of cancer-related fluid within the compartment between the lung and chest wall (the pleural space), resulting from metastasizing cancer cells, extravasating the bloodstream or lymphatic system and invading the pleural space, leading to fluid accumulation. The American Thoracic Society have published statistics showing that 2%–12% of all patients with breast cancer will develop a malignant pleural effusion and 36%–65% of cases are affected in cases of disseminated disease or at autopsy (American Thoracic Society, 2000). Meanwhile, subsets of metastatic breast cancer patients have an organ specific predilection for the pleural space (Soni et al., 2015). As many as 1 million individuals per year are estimated to be affected by MPE worldwide (Piggott et al., 2023) and breast cancer is superseded only by lung cancer as the underlying etiology (Han et al., 2024). Patient quality of life is poor and median survival time is 6–18 months for breast-derived MPE (Zamboni et al., 2015; Fentiman et al., 1981; Wang Y. et al., 2023). Clinical presentation of MPE most frequently involves dyspnea, impaired chest wall movement and dysfunction of the diaphragm (Munavvar et al., 2025). These frequently debilitating symptoms add further to the discomfort and psychological suffering of an already mentally and physically burdensome disease (Zaza and Baine, 2002; Guo et al., 2023). The tumor spread underlying MPE pathogenesis is in part promoted by the dysregulated formation of leaky tumor neo-vasculature that is part of metastatic cancer pathogenesis (Economidou et al., 2010; Jovanovic, 2021). Tumor-driven vascular growth factors (Jovanovic, 2021) and a purported cancer promoting pleural immune environment (Donnenberg et al., 2023) are driving factors for malignant progression and often voluminous fluid accumulation.

### 1.1 Standard-of-care

In the Western world, MPE is regarded as a resulting from the end stage of an incurable malignancy. Driven by a belief in the inevitability of finality and by healthcare directives frequently concerned with the optimal economics of care, treatment is in many instances is directed predominantly to symptomatic, mechanical palliation (Sivabalah et al., 2024). While the ultimate goal is to reduce physical symptoms, economics are a central consideration, with efforts made to minimize hospital stays and treatment costs (Munavvar et al., 2025; Shafiq et al., 2015; Siefen et al., 2023). Palliative strategy education efforts seek to inform oncologists of the best effusion management strategies as developed by the American Thoracic Society (ATS) the Society for Thoracic Surgeons (STS) and the European Association for Cardio-Thoracic Surgery (EACTS) among others (Duong et al., 2025). While post-treatment quality-of-life measures have been considered and integrated within treatment strategies, attempts to measure psychosocial impacts of mechanical treatments have been inconsistent (Peel and Mishra, 2023). Furthermore, patients are predisposed to multiple complications including discomfort or infections that have been historically under-recognized but have physical and psychological implications that negatively impact quality-of-life (Iqbal et al., 2024).

Despite clearly formulated guidelines (Duong et al., 2025), palliative treatments can be applied inconsistently depending on location, local expertise or a physician’s experience (Sidhu et al., 2024; Mei et al., 2023). The lack of effective or perceived effective systemic therapy strategies for MPE leaves the management of these suffering patients most often to surgical services or interventional radiology, which can be superb in centers of excellence and potentially deficient in “safety-net” hospitals (Sarkar et al., 2020; Thomas et al., 2014). While some advances have occurred in recent years, these mechanical treatments remain frequently painful, body image altering and potentially morbid (Munavvar et al., 2025; Gonnelli et al., 2024). Although well-trained thoracic surgeons are skilled in palliative mechanical solutions for MPE, enhanced systemic or regional therapies may offer the possibility of MPE control while better addressing underlying disease and would therefore be welcomed (Thomas et al., 2014). Amongst cancer patients and care providers there has been long-standing recognition that treatment should be personalized to the patient and consider wide-ranging individual factors (Thomas et al., 2014; Orlandi et al., 2024).

A primary mechanical treatment approach which has recently gained traction is the placement of an indwelling pleural catheter (IPC), which is selectively applied based on lung expansibility and characteristics of the patient or effusion (Boshuizen et al., 2013; Bhatnagar et al., 2015). Here, a catheter is tunneled under the skin and into the intrapleural space. This allows individuals to undergo continuous or intermittent drainage in an ambulatory home environment (Thomas et al., 2014; Semenova et al., 2025). The use of IPC has been shown to be successful at reducing symptoms and is often touted as patient-centric (Mitchell et al., 2023) but despite the apparent convenience of ambulatory repeat drainage-on-demand, multiple undesirable side effects have been reported. These include IPC-related infections, IPC-site metastasis, pain, itching, pleural fluid loculation, and issues with the IPC itself including catheter blockage and IPC fracture leading to a catheter fragment being retained (Iqbal et al., 2024; Wang S. et al., 2023). Furthermore, patients report negative impacts on their wellbeing and quality-of-life that include anxiety and altered relationships (Peel and Mishra, 2023; Zhang et al., 2023).

A commonly employed alternative approach recommended by expert organizations is pleurodesis. This amounts to a physical obliteration of the intrapleural space by surgically administered, abrasive or inflammation-inducing agents such as dry talc or talc slurry between the pleura (Orlandi et al., 2024; Kathamuthu et al., 2025). First described by Norman Bethune in 1935 (Munavvar et al., 2025; Thomas et al., 2014) chemical pleurodesis remains a common course of treatment in the modern era. One recent study reported that 22.7% of MPE patients were treated by pleurodesis while 77.3% received IPC, usually significantly longer after cancer diagnosis (Kwok et al., 2024). While literature has been dedicated to the overall safety of pleurodesis (Baiu et al., 2020), it is nonetheless associated with a range of undesirable side-effects that include pain, fever, dyspnea, pneumothorax and pneumonia at incidences ranging from 4% to 20%, as well as rare events such as respiratory failure and acute respiratory distress syndrome (Zhang et al., 2021).

It is prudent to reiterate the mechanical palliative nature of these mainstay treatments. Beyond the physically destructive elements of pleurodesis, or the physical and psychosocial ill-effects of IPC, neither attempt to address the underlying malignancy nor are expected to extend the lifespan of the patient. However, a striking disparity in treatment patterns exists worldwide. While Western recommendations appear to focus on mechanical palliative solutions, international practices include novel regional approaches (Chinese Thoracic Society and Chinese Medical Association, 2023; Xu et al., 2024).

### 1.2 Alternatives to standard-of-care

Beyond the West, in an era of personalized medicine and targeted tumor treatments, modernized approaches to treat breast MPE are routinely employed or under active investigation. International clinical trials involving MPE patients routinely describe treatment approaches that involve pharmaceutical agents administered intrapleurally and designed to physically target the tumor or the neovascular network responsible for the effusion. Many studies describe treatment of breast or lung cancer-driven MPE using an anti-vascular agent based on a modified Rh-endostatin (Endostar), frequently combined alongside intrapleural chemotherapy (Wang et al., 2021; Fan et al., 2018; Biaoxue et al., 2016). Endostar was approved by the China State Food and Drug Administration for the treatment of non-small cell lung cancer as the first-line therapy in 2005 (Biaoxue et al., 2016). Despite widespread reports of successful combined cancer treatment and management of MPE symptoms, we are aware of no studies that directly compare the efficacy of these Endostar-based treatments to Western mechanical palliative approaches.

While intrapleural Endostar and combined chemotherapy appear to be unique as a commonly adopted first line alternative, the concept of intrapleural tumor or vascular targeting treatments is not unique and has wide and long-standing precedent. Examples in animal models and human clinical trials exist of intrapleural administration of bevacizumab, bispecific antibodies, immunotherapeutics and other wide-ranging approaches (Hao et al., 2025; Khosrawipour et al., 2023). In fact, intrapleural administration of the bispecific antibody catumaxomab was tested in an phase 1/2 trial in MPE patients and 5 of 7 evaluable breast cancer patients showed positive response to the treatment at day 60 post infusion (Sebastian et al., 2009). One patient had complete response, defined as relief of symptoms related to the effusion with absence of fluid reaccumulation. The remaining four patients had partial response, defined as diminution of dyspnea, with partial reaccumulation of fluid and no further therapeutic thoracenteses required (Ammouri and Prommer, 2010). It should be noted however that this study included 17 non-breast cancer patients with MPE, two of whom experienced dose-limiting toxicities, with one ultimately passing away from symptoms believed to be caused by the treatment. In a separate Phase I study (Aggarwal et al., 2018) a breast MPE patient treated with gene-mediated cytotoxic immunotherapy achieved stable disease and the treatment was safe and well tolerated in a cohort of MPEs arising from various primary cancers. Another phase I study (RIOT-2) is currently underway to assess the use of intrapleural or intraperitoneal tocilizumab in patients with MPE secondary to any metastatic cancer (Park et al., 2024). The case for intrapleural administration of immunotherapies to treat MPE has been extensively reasoned by others (Donnenberg et al., 2019), and intrapleurally administered anti-PD1 antibody controlled malignant pleural effusion and the growth of cancer when tested in murine models of lung MPE (Li et al., 2021). De-platinum-based pleural perfusion bevacizumab was shown to be efficacious in managing MPE in lung cancer patients (Wang P. et al., 2024). Other agents tested intrapleurally in lung cancer include bispecific antibodies (Kroesen et al., 1997; Cai et al., 2024), bevacizumab, anti-angiogenic tyrosine kinase inhibitors, cytokine-based immunotherapy, tumor necrosis factor-α treatment, intrapleural immunogene therapy, tumor infiltrating lymphocyte treatment and more (He et al., 2021). A recent review states that bevacizumab and Endostar have been approved for MPE treatment, although we have been unable to verify this independently (Xu et al., 2024). Nonetheless, much of the prior work described has been conducted in very limited cohorts or is preliminary, indicating a need for more extensive, robust and disease specific future studies.

While encouraging preliminary reports of efficacy have been described and reviewed in other works (Murthy et al., 2019; Wong et al., 2023), it is our opinion that these modernized pharmaceutical approaches to MPE treatment have been significantly understudied. There appears to be a dearth of Western studies investigating these alternative regional treatment modalities (He et al., 2021). We believe this is partially due to the widely recommended approach from medical societies to apply mechanical treatment as first line (Munavvar et al., 2025; Sivabalah et al., 2024; Duong et al., 2025), but is also influenced by unavailability of Endostar (Wang D-X. et al., 2024) and local recommendations against routine use of intrapleural treatment by official bodies, despite the recognition that early evidence may support improved effusion control and quality of life through intrapleural combination therapies (Sivabalah et al., 2024).

Of course, such recommendations are not without reason. MPE patients are at a stage of advanced disease and will frequently be frail. Attempts at novel treatment are not without danger, and serious toxicities or occasional deaths have been reported, as previously discussed. Furthermore, standard-of-care approaches, including systemic treatments are familiar to clinicians and are often well-tolerated by patients. For clinical staff with a patient’s wellbeing as a primary consideration, accepting the uncertainties of intrapleural treatments could be considered an unacceptable risk even where recognition of the potential of a novel treatment is recognized and appreciated.

The lack of research in this area is potentially exacerbated by challenges in clinical trial recruitment due to MPE patients’ frequently poor performance status not meeting inclusion criteria (Penz et al., 2017). Furthermore, understanding of the processes underlying metastasis to the pleural space and effusion development are incomplete (Penz et al., 2017) and assessment of new cancer treatments is in general impeded by the challenges in human testing and a lack of readily employed or representative models of tumors and their microenvironment (Honkala et al., 2022). Nonetheless, despite these challenges we believe the potential offered by further exploration of intrapleural treatment options is significant, particularly when patient-derived samples from MPE are abundant, can be obtained in a minimally invasive manner, and are already collected as standard-of-care. Such rich sampling of what may represent the most malignant compartment of a patient’s disease provides a basis for *ex vivo* drug sensitivity testing on a broad scale. A biomimetic testing platform could offer the ability to profile a broad armamentarium of therapeutic agents and significantly de-risk the administration of novel therapies in instances where standard of care is deemed insufficient.

While animal models have existed for some time, they are laborious and slow to test. Further, these may be inadequately representative of the patient tumor, and are increasingly falling out of favor (Mak et al., 2014) as concerns about ethical treatment of animals grow, to the extent that the NIH have stated they will no longer directly support animal model-only research (NIH, 2023).

### 1.3 Functional precision medicine in cancer

Functional precision medicine involves direct exposure of patient-derived tissues to drugs in order to attempt prediction of clinical response (Letai, 2017). The concept is not new, with a history extending more than 7 decades. Perhaps the first report of using patient-derived primary tumor tissues to predict chemotherapy response *ex vivo* was the work of Dr. Jane Wright, a founding member of the American Society of Clinical Oncology, in 1957 (Napoli et al., 2022). Pioneering chemosensitivity assays appeared in the late 1970s, primarily based on clonogenic tumor properties, with numerous assays developed since and culture methods and readouts varying across time (Morand du Puch et al., 2021). Some seminal efforts described the use of microtiter plate assays to evaluate immunological and chemical sensitivity of tumors to various agents (Kornblith, 1978). Many methods brought initial promise but were ultimately later eliminated (Su, 2014) for various reasons. For example, clonogenic assays required long assay times and large numbers of cells for limited drug evaluations, while exhibiting growth artifacts due to minor culture plate manipulations and irreproducibility of result between labs, among many other listed shortcomings (Weisenthal and Lippman, 1985). Editorials from the New England Journal of Medicine and American Society for Clinical Oncology cast doubt on the ability of the assays to personalize care (Letai et al., 2022). Problems included low evaluation rates (Su, 2014), variability in responses, lack of reproducibility (Napoli et al., 2022), labor-intensiveness, interpretability issues, need of highly skilled operators (Su, 2014), long turnaround times, poor scalability (Crystal et al., 2014; Friedman et al., 2015) and lack of translatability to clinical response (Morand du Puch et al., 2021). To our knowledge, these editorials did not propose alternative testing strategies, but rather focused on describing fundamental limitations of the available assays and therapeutic context at the time. Several commercial tests have subsequently become available (Napoli et al., 2022) but ultimately a protocol is yet to be unanimously recognized by the biomedical community or the regulatory authorities (Morand du Puch et al., 2021). Thus, no methods are widely accepted by the medical community (Su, 2014).

### 1.4 Modernization and automation

It has been widely reported that traditional 2D chemosensitivity testing fails to recapitulate the *in vivo* environment in which a tumor evolves and it has been stated that an ideal model should mimic this environment as well as the tumor genomics (Napoli et al., 2022). Traditional 2D techniques have been shown to cause cytoskeletal remodeling, and altered gene and protein synthesis (Knight and Przyborski, 2015). Studies in such cell cultures have shown cells progressively flatten and lose their differentiated phenotype (Petersen et al., 1992; Fuchs et al., 2004), therefore lacking accurate tissues architecture, cellular interplay and personal heterogeneity aspects (de la Puente et al., 2015; Fan et al., 2019). They also fail to reproduce nutrient and oxygen gradients, which impairs the ability to precisely predict drug activity (Santo et al., 2017). 2D culture lacks natural extracellular matrix (ECM) proteins, chemokines, growth factors and sites for cellular adhesion, which are crucial for cells to interact with adjacent cells and surroundings, preserving the specificity and homeostasis of the original tissue and its regular functioning (Kleinman et al., 2003; Riedl et al., 2017).

For some time, the field has been transitioning toward powerful alternative models that could facilitate testing of novel treatment methods utilizing patient-derived systems that represent the individual’s tumor more closely and readily than any traditional approach. 3D patient-derived organoids maintain various features of the original tumor like intratumoral heterogeneity, secondary architecture, and polyclonality and are seen as a means of overcoming many traditional shortcomings (Napoli et al., 2022).

Automation has the ability to address multiple issues inherent in traditional and manual assays. Variability in culture experiments and even in organoid assays are well recognized as problematic. Disparities in cell procurement, sample storage, media composition and downstream experimental protocols are only a few examples of considerations that have the potential to introduce experimental variability (Tong et al., 2024).

It is accepted that industrial developments including automation and miniaturization will be at the core of addressing many challenges associated with traditional assays (Taurin et al., 2025; Zhao et al., 2022; Wensink et al., 2021; Jiang et al., 2020; Yang et al., 2025). Prior publications have identified assay variability issues due to extracellular culture matrices (Zhao et al., 2022; Yang et al., 2024; Aisenbrey and Murphy, 2020; Li et al., 2025; Lumibao et al., 2024; Driehuis et al., 2020), manual handling inconsistencies (Jiang et al., 2020), and media variability (Zhao et al., 2022; Driehuis et al., 2020). Automation will also facilitate clinically relevant turnaround times. These are key to any assay that will be adopted widely (Foo et al., 2022), with the success of patient-derived organoid-based drug screening in personalized cancer care being dependent on rapid turnaround from tumor sampling to drug recommendation to guide treatment decisions in a clinically relevant timeframe (Yang et al., 2024; Xiang et al., 2024).

### 1.5 Modern approaches to breast MPE

Several studies in recent years have described success in establishing patient-specific organoids from MPE underlying breast cancer (Chang et al., 2024). A summary of traditional and modern approached to MPE care is provided in Table 1. Organoids have been reported to retain the histological features, receptor status and the hotspot mutations of the parent cancer (Önder et al., 2023). Immune cell subtypes have also been shown to be similar between breast primary tumor and breast MPEs in pilot studies (Laberiano-Fernandez et al., 2024). Furthermore, dose response testing has been shown to mirror the drug sensitivity profile of the patient (Pan et al., 2021; Choi et al., 2023). Collectively this preliminary work suggests potential applications for functional precision medicine in advancing care of breast MPE. The promise of organoid technology has been further evidenced and bolstered by the recent establishment of the NIH Standardized Organoid Modeling (SOM) Center with the goal of developing standardized organoids and protocols for biological and medicinal research, with $87 million in contracts to awarded in its first 3 years (NIH, 2024).

**TABLE 1:** A summary of traditional, contemporary and investigational approaches to treatment of malignant pleural effusion.

Approach	Mechanism	Advantages	Limitations
Indwelling pleural catheter (IPC)	Continuous or intermittent mechanical drainage of pleural fluid via a catheter inserted into the intrapleural space	Offers effective symptom palliation, enables outpatient management, is widely available and well characterized	Does not target underlying malignancy and carries risks including infection, catheter malfunction, pain, and negative psychosocial impact. Does not address tumor biology and is not designed to confer survival benefit from underlying disease
Chemical pleurodesis	Induction of inflammation and fibrosis with chemical agents to cause obliteration of the pleural space and prevent reaccumulation of fluid	Can reduce recurrence of pleural effusion and is a single intervention procedure	Requires inpatient care. Is associated with potential for pain, fever, dyspnea, pneumothorax, and rare respiratory complications. Does not address tumor biology and is not designed to confer survival benefit. Success rates more variable than IPC.
Intrapleurally administered chemotherapy or anti-angiogenic therapy	Regional delivery of cytotoxic or vascular-targeting agents directly to the pleural space	High local drug concentration and reduced systemic exposure with the potential for improved effusion control. Has potential for local disease control	Limited Western study or adoption currently, with heterogeneous protocols in small clinical cohorts. Lacks direct comparison to traditional mechanical approaches currently
Functional precision medicine guided intrapleural therapy	Functional drug testing of patient-derived pleural tumor cells potentially combined with omics profiling to inform therapeutic selection	Has the potential to enable patient-specific treatment selection using effective localized targeted therapies that directly address the tumor biology, treating symptoms while potentially prolonging life	Still early-stage and investigational. Variable organoid establishment rates to-date. Will require study and clinical validation

Next-generation sequencing (NGS) approaches have also been promoted as high value in MPE profiling and offer diagnostic, prognostic and theranostic value. Concordance of mutations between lung cancer MPE and primary tumor samples using NGS has been high and it is reported that studies in breast and other cancers have also shown promising results. For example, targeted deep sequencing of cell-free DNA from predominantly pleural effusions demonstrated high concordance with matched tumor tissue sequencing, enabling detection of previously identified driver mutations as well as new variants likely associated with acquired drug resistance (Yang et al., 2020). The versatile applications of NGS open avenues to ready assessment of tumor content, genome-wide mutation profiling, neoantigen detection and more. Via these methodologies, assessments of the MPE compartment’s genomic landscape versus that of a primary tumor can be assessed to ensure adequate tumor cell presence and genetic similarity. While treatments can sometimes be targeted to known genes or mutations individually, combination with a functional assay is imperative prior to taking on potentially risky or expensive regional therapies (Liu et al., 2022). NGS likely represents a high-value partner assay to functional readouts that directly monitor drug responsiveness, including organoids or similar platforms.

**FIGURE 1:**
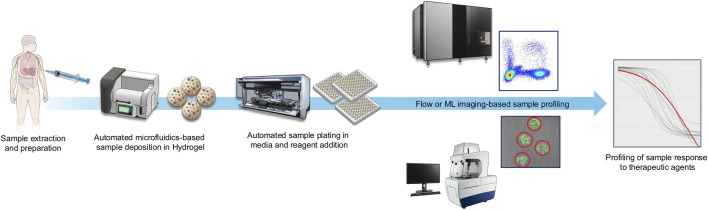
Schematic of our workflow. Samples are acquired from a patient and deposited in uniform, microfluidics-created, spherical hydrogel scaffolds with proprietary technology. Automated processing enables deposition of these in reaction plates with media where they can be maintained, profiled and drug treated. Cell populations are assessed by spectral flow cytometry and cytometry-based cell counts can be utilized in determining cellular response to drug addition. Widefield microscopy combined with machine-learning based analysis can image, identify and measure signals of cell viability to determine drug-sensitivity based on markers of interest, while simultaneously identifying morphological characteristics of cells and heterogeneity of response.

Utilizing a proprietary microfluidic 3-dimensional organoid technique (Wang et al., 2022), we have observed promising early results suggesting the feasibility of up-scaling functional precision medicine in the context of solid (Ding et al., 2022a; Chakraborty et al., 2023; Köhler et al., 2025; Gobits et al., 2025) and liquid tumors (Xi et al., 2023a; Xi et al., 2023b) including breast cancer (Graham et al., 2023) and doing it in clinically relevant turnaround times of approximately 7 days. Our push-button automated MOSgen™ instrument automatically encapsulates cells present in a fresh patient sample to generate MicroOrganosphere (MOS®) droplets and is compatible with downstream analysis by flow cytometry or brightfield microscopy combined with fluorophore-conjugated antibody staining and combined with computational imaging analysis (Figure 1). Tumor and key functional components are collectively encapsulated in hydrogel droplets which are solidified through exposure to electronically controlled light or temperature changes (Wang et al., 2022). In preliminary and published works, we have demonstrated the feasibility of retaining stromal and functional immune microenvironment components, and demonstrated the effects of immuno-oncology agents (Ding et al., 2022a; Ding et al., 2022b). Immune-tumor cell interactions can be optionally observed utilizing longitudinal imaging, depending on study requirements. NGS can be utilized as required by a study (Ding et al., 2022a; Ding et al., 2022b), pre or post droplet generation.

Using our approach, our latest published manuscript demonstrated 83% accuracy in a cohort of 21 neoadjuvant standard-of-care treated colorectal cancer (CRC) patients in a retrospective clinical study and further possessed discriminative ability for responsive versus non-responsive patient-derived tumor models using disease-free survival as a clinical endpoint (Gobits et al., 2026). For each patient samples approximately 40 single-patient-derived organoids were processed per well in 384 well plates covering multiple therapeutic agents with 9 dose gradients and 4 replicates per-dose. We demonstrated clinically relevant assay turnaround times (∼7 days) and furthermore demonstrated high reproducibility among control samples (coefficient of variation range 1.7%–2.5%). In this recent study, NGS was invaluable yet primarily utilized in order to identify presence and quantity of known CRC mutation patterns for confirmation of tumor identity in organoids, however our future plans as they correspond to MPE would differ by actively profiling for drug sensitivity markers in a prospective fashion. We believe that the described characteristics of our system as well as specific published evidence provide indications of having overcome multiple historical shortcomings of traditional functional precision medicine models described in Section 1.3 above, including lack of translatability to clinical response, long turnaround times, poor scalability, lack of reproducibility, and reduced need for highly skilled operators.

We have preliminary experience in generating models from breast MPE samples in early exploratory work. Published studies from other groups report establishment success rates of 20%–33% (Pan et al., 2021; Choi et al., 2023). Our own initial efforts support the feasibility of establishing patient-derived tumor models from MPE samples. Early model characterization work indicates we retain key features of the parent sample, including presence of tumor and CD45^+^ immune cells. In these experiments, early observations suggest retention of key immune components including helper and cytotoxic T-cells, as well as a non-T cell niche, and indicate preservation of relative cell proportions across periods of up to 7 days. Our past successes in other disease areas make us hopeful that future efforts will enable us to expand upon the promising organoid work reported by others, while taking advantage of our technology’s scalability and automation. Our prior peer-reviewed work in other disease types has demonstrated turnaround times compatible with clinical treatment selection, as well as correlation between patient-derived organoid drug responses and clinical response in retrospective studies (Wang et al., 2022; Ding et al., 2022a; Gobits et al., 2026), with further peer-reviewed studies currently in press. Collectively these findings add confidence that we will be able to successfully deploy clinically valuable predictive assays in breast MPE in imminent future work. We plan to actively devote efforts to this nascent area of research, while encouraging others in the field to consider similarly expanding their own efforts, based on the patients’ need and the rich possibilities for improved treatment described in this perspective.

### 1.6 Discussion

For some time now, the field of oncology has benefited from an era of personalized treatment based on predictive precision medicine approaches. Functional precision medicine technologies such as organoids increasingly offer the added possibility of treatment based on actual observed drug effects in model systems representative of patient tumor genetics and biology. To date, the standard-of-care for breast MPE has not meaningfully benefited from these modern paradigms, but several characteristics of the disease make it an excellent candidate for increased study. MPE yields relatively large numbers of tumor cells, in contrast to solid metastases, where cell numbers are fewer and biopsy is frequently risky. Since MPE fluids are removed for palliative care, no additional intervention is required and risk to the patient is reduced. This large volume of tumor cells from MPE enables direct interrogation of drug response without the need to expand small amounts of tumor cells and increase risk of clonal selection. This also increases speed of processing so that a treating physician may render a decision quickly, and limit drift in the populations of cells beyond the tumor cells alone.

As we have described, generic organoid studies have shown significant initial promise in the profiling and treatment of breast MPE, but the approach is inherently limited. The tumor microenvironment is increasingly appreciated as a key player in the tumorigenesis and disease progression (Hanahan, 2022). Furthermore its role in regulating treatment response is progressively being understood, particularly with regard to adaptive cell responses and the part they play in immune checkpoint blockade response and resistance (Aliazis et al., 2025; Wang Q. et al., 2023). Despite some success, a central hurdle to successful clinical implementation of traditional organoid technologies has been the lack of a representative tumor microenvironment (Thorel et al., 2024; Zhou et al., 2023). A solution that maintains both tumor cells and key functional components of the tumor microenvironment has the potential to more closely mimic cancer biology, replicate treatment effects *ex vivo* and open new avenues of clinical utility.

We believe that in our MOS platform we currently possess technology and techniques that enable large-scale testing of breast MPE models with replication of tumor phenotype and microenvironment (Wang et al., 2022; Chakraborty et al., 2023; Graham et al., 2023; Ding et al., 2022b; Gobits et al., 2026). Furthermore, the platform’s automated and microfluidic-controlled deposition of a primary cellular tumor sample in uniform 3D hydrogel spheres, reduces processing times from weeks or months (Huang et al., 2024; Tzeng et al., 2023) to days (Wang et al., 2022; Ding et al., 2022a; Chakraborty et al., 2023; Graham et al., 2023). Ultimately this offers the potential for *ex vivo* testing at new levels of scale and translatability. Our approach involves all the components necessary to enable the identification of precision medicine targets in the application of existing agents, and to enable discovery and development of entirely novel therapeutics. While breast MPE represents just one disease suited to application of our technology, it is our goal to imminently expand study in this area to drive clinical benefit for what we consider a patient population with significant potential for improved care. We look forward to a period of combined precision and functional precision medicine where ourselves and our colleagues in the field can realize the potential of the latest technological advances to reimagine treatment paradigms in breast cancer-related MPE, along with other underserved disease areas where standard-of-care has stagnated, and the potential for improved care is significant.

## Data Availability

The raw data supporting the conclusions of this article will be made available by the authors, without undue reservation.
